# Prevalence of HIV-related pain in Japan: a clinical survey

**DOI:** 10.1007/s00540-025-03493-y

**Published:** 2025-04-16

**Authors:** Megumi Kanao-Kanda, Sarah Kyuragi Luthe, Yoshiko Onodera, Izumi Sato, Tomoyuki Endo, Tomoyuki Kawamata, Hirotsugu Kanda

**Affiliations:** 1 Department of Anesthesiology, Wakayama Medical University School of Medicine, 811-1 Kimiidera, Wakayama, Wakayama 641-8509 Japan; 2 Department of Anesthesiology and Critical Care Medicine, Asahikawa Medical University, Midorigaoka-Higashi 2-1-1-1, Asahikawa, Hokkaido 078-8510 Japan; 3 Department of Hematology, Hokkaido University, Kita 14-Nishi 5, Sapporo, Hokkaido 060-8648 Japan

**Keywords:** HIV, Neuropathic pain, Clinical survey

## Abstract

**Background:**

Although human immunodeficiency virus (HIV)-related peripheral neuropathies are among the most common neurological complications in patients with HIV infection, the prevalence and patient characteristics of HIV-related pain and peripheral neuropathic pain in Japan remain unclear.

**Objectives:**

This study aims to investigate the prevalence and patient characteristics of HIV-related pain with a focus on peripheral neuropathic pain among Japanese patients.

**Methods:**

We conducted a survey among patients diagnosed with HIV infection and reviewed their medical records to collect the following information; age, sex, presence of pain or numbness, duration of pain or numbness, duration of HIV infection, clusters of differentiation 4 (CD4) T-cell count, ribonucleic acid (RNA) load, diagnosis and duration of acquired immune deficiency syndrome (AIDS), treatment status and duration of highly active antiretroviral therapy (HAART). The primary outcome of this study was the prevalence of HIV-related pain with a focus on peripheral neuropathic pain.

**Results:**

A survey was distributed to 474 patients, of whom 270 chose not to participate. Consequently, data from 204 patients were included in the analysis. The prevalence of HIV-related pain was 16% and patients with possible HIV-related peripheral neuropathic pain was 9.3%. Among these patients, age, presence of numbness, duration of numbness, and duration of AIDS were significantly higher than in patients without HIV-related pain.

**Conclusions:**

In this prospective multi-center cross-sectional study, the prevalence of HIV-related pain was 16% among 204 Japanese patients with HIV in which they tended to have advanced age and longer duration of AIDS compared to patients without HIV-related pain.

## Introduction

Human immunodeficiency virus (HIV) is a well-documented condition in the United States, affecting over 1.2 million people nationwide [1]. Among the numerous complications associated with HIV, HIV-related pain is one of the most commonly reported symptoms in people living with HIV [2], although prevalence estimates vary widely. One systematic review reported a point prevalence of 54–83% using a three-month recall period [3]. In contrast, the cumulative total of people living with HIV in Japan was reported to be 23,863 in 2021 [4], yet HIV-related pain remains underrecognized among both Japanese patients and healthcare providers. Furthermore, chronic pain is prevalent among individuals with HIV [5], which can lead to significant disability and poor outcomes [6].

Common types of HIV-related pain include neuropathic pain, musculoskeletal pain, headaches and abdominal pain [7]. Among these, HIV-related peripheral neuropathy is the most common neurological complications in patients with HIV infection [8–10], with an estimated prevalence of 6.9–10% in a systematic review of epidemiological studies [11]. HIV-related peripheral neuropathy can affect both sensory and motor nerves in the distal limbs, leading to clinical manifestations such as pain, numbness, loss of sensation, paresthesia, burning sensation, and stabbing sensations. These symptoms typically present in a stocking-glove distribution primarily affecting the feet and hand, and can be profoundly debilitating for patients [10, 12]. In reference to the underlying neuropathological mechanisms of HIV-related peripheral neuropathy and its treatment, our team has utilized viral vectors in animal experiments to suggest that targeting mitochondrial dynamics may be beneficial [13–15]. However, the exact mechanism remains unknown. In addition, although early initiation of highly active antiretroviral therapy (HAART) can benefit in preventing neurological complications with HIV infection, distinguishing between HIV-related peripheral neuropathy caused by HIV itself, and the neurotoxic effects of HAART, known as antiretroviral toxic neuropathy (ATN) [16], remains challenging. Accordingly, reported risk factors for HIV-related peripheral neuropathy include treatment with HAART, low clusters of differentiation (CD) 4 count, elevated plasma HIV-1 RNA viral load, advanced age, and history of substance abuse [17]. A nationwide survey conducted in Japan before the introduction of HAART in 1996, showed that 2.6% of the 578 individuals with AIDS reported peripheral neuropathy [18]. However, the prevalence and characteristics of HIV-related pain and peripheral neuropathy in Japan remains unclear. Therefore, we aimed to evaluate the characteristics and prevalence of patients with HIV-related pain in Japan with a focus on peripheral neuropathic pain.

## Patients and methods

### Study design

We conducted a prospective multi-center cross-sectional study. This study was approved by the Asahikawa Medical University Research Ethics Committee (approval number: 16215) and Hokkaido University (Independent Clinical Research Number: 017-0061) and was registered as a clinical trial (UMIN 000036713). Written informed consent was obtained from all patients.

### Inclusion criteria

We included patients with HIV infection who visited the Department of Hematology at Hokkaido University Hospital and the HIV outpatient clinic at Asahikawa Medical University Hospital for treatment or follow-up between April 18, 2017 and May 11, 2021. We excluded patients with cognitive impairment due to mental disorders or central nervous abnormalities, those who demonstrated non-compliance with the survey, and those with incomplete medical records. Consequently, we distributed the survey to 474 patients, of whom 270 chose not to participate. Finally, data from 204 patients were included in the analysis.

### Data collection

We conducted a survey among patients diagnosed with HIV infection in addition to reviewing medical records to collect the following information regarding each patient; age, sex, presence of pain or numbness, duration of pain or numbness, duration of HIV infection, CD4 T-cell count, ribonucleic acid (RNA) load, diagnosis and duration of acquired immune deficiency syndrome (AIDS), treatment status and duration of HAART. The clinical survey was based on common symptoms associated with HIV-related pain and peripheral neuropathic pain and by evaluating questionnaires that were previously used to assess pain, including the Japanese version of the “painDETECT” questionnaire [19] referenced from the executive summary of clinical guidelines of pharmacotherapy for neuropathic pain provided by the Japanese society of Pain Clinicians [20]. The primary outcome of this study was the prevalence and characteristics of patients with HIV-related pain and peripheral neuropathic pain. Table 1 displays the Japanese survey translated into English.

**Table 1:**
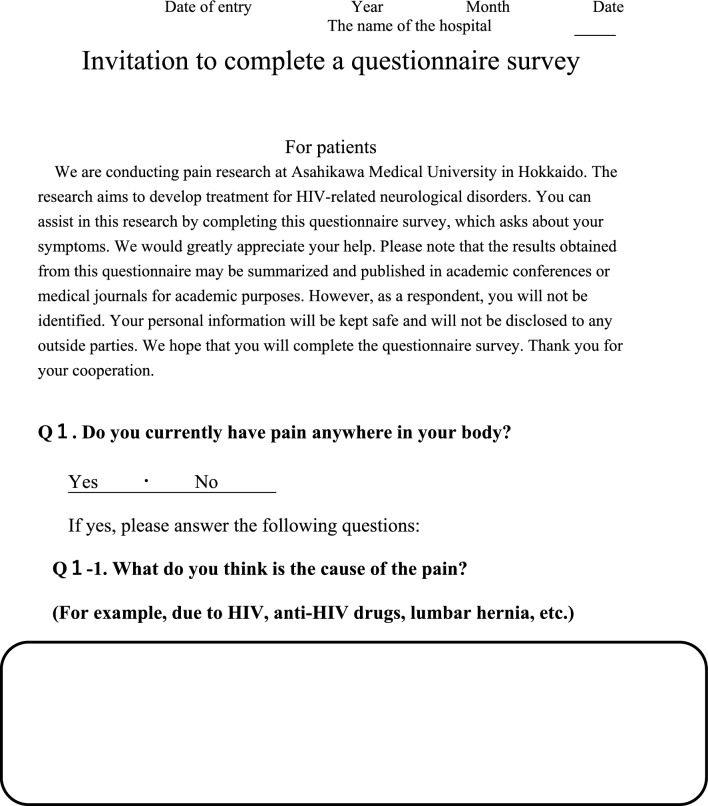

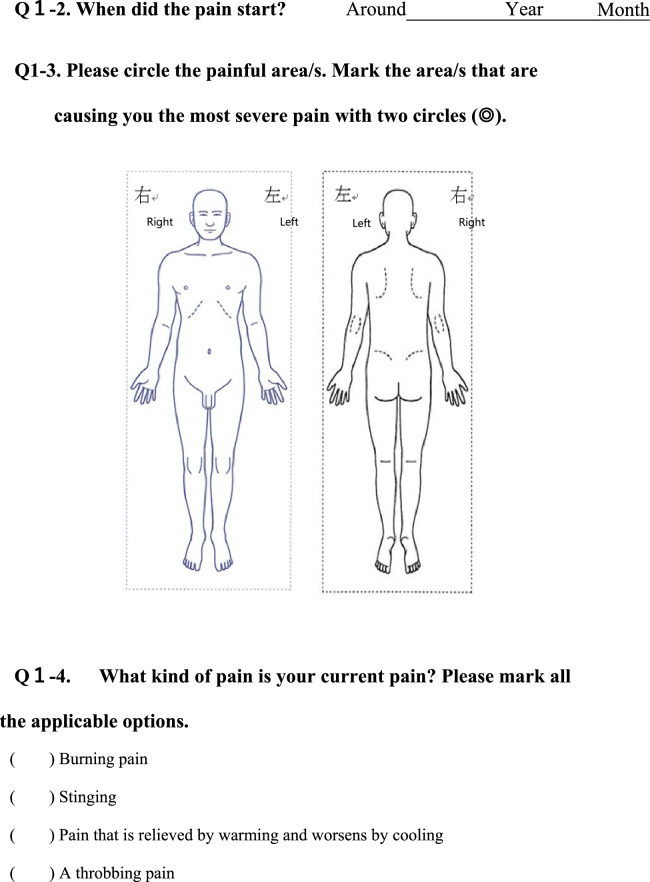

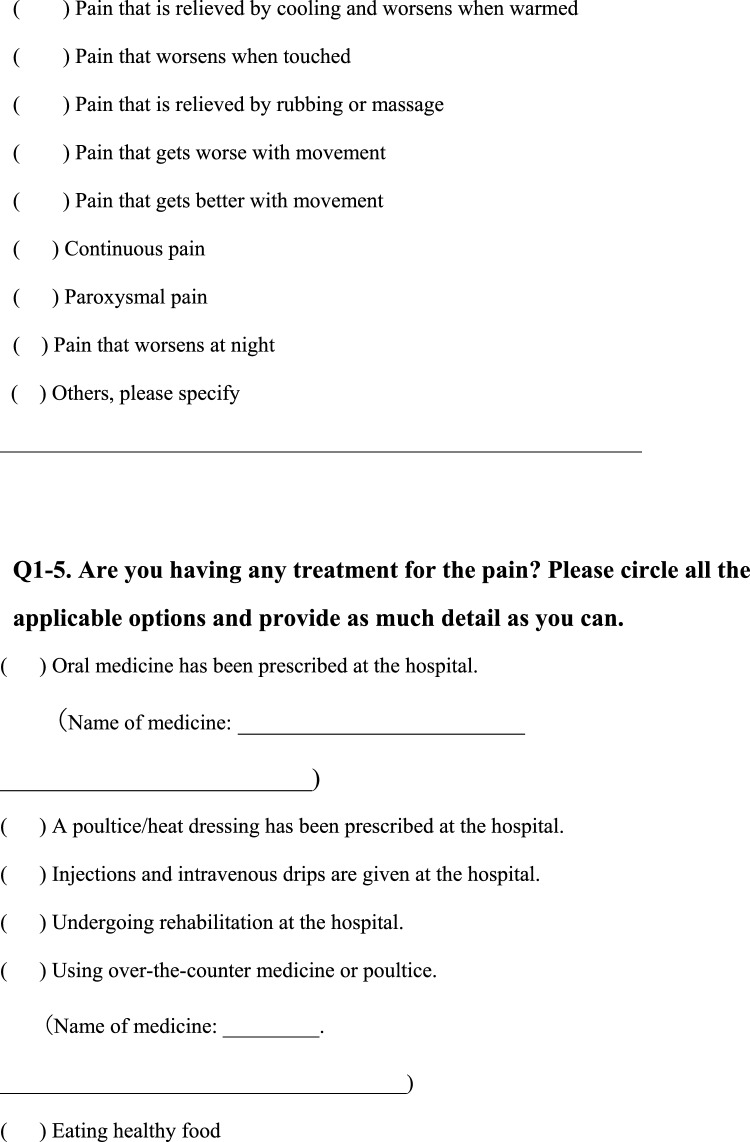

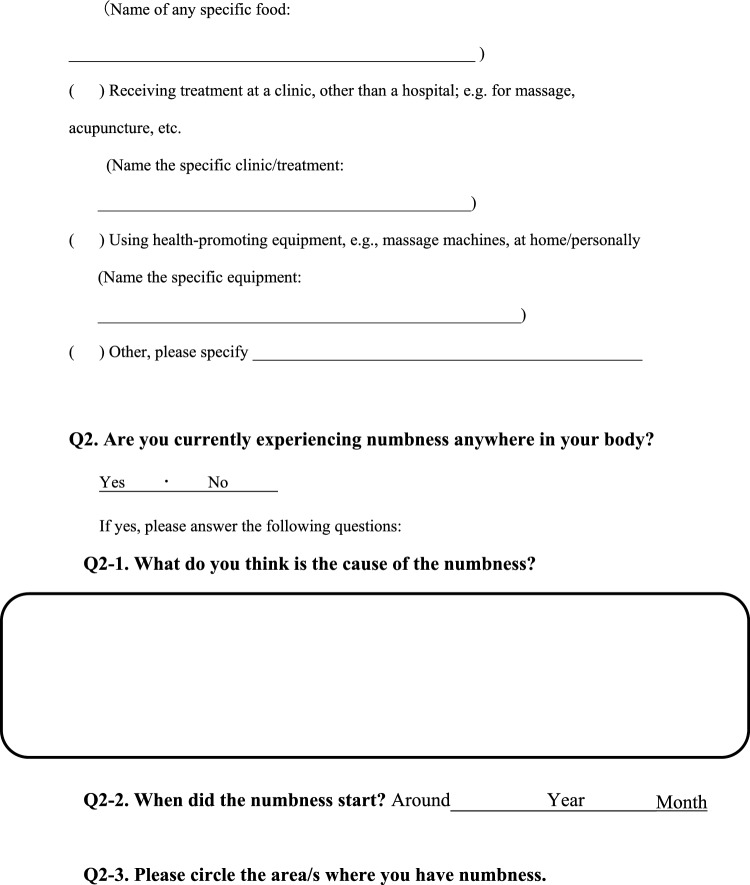

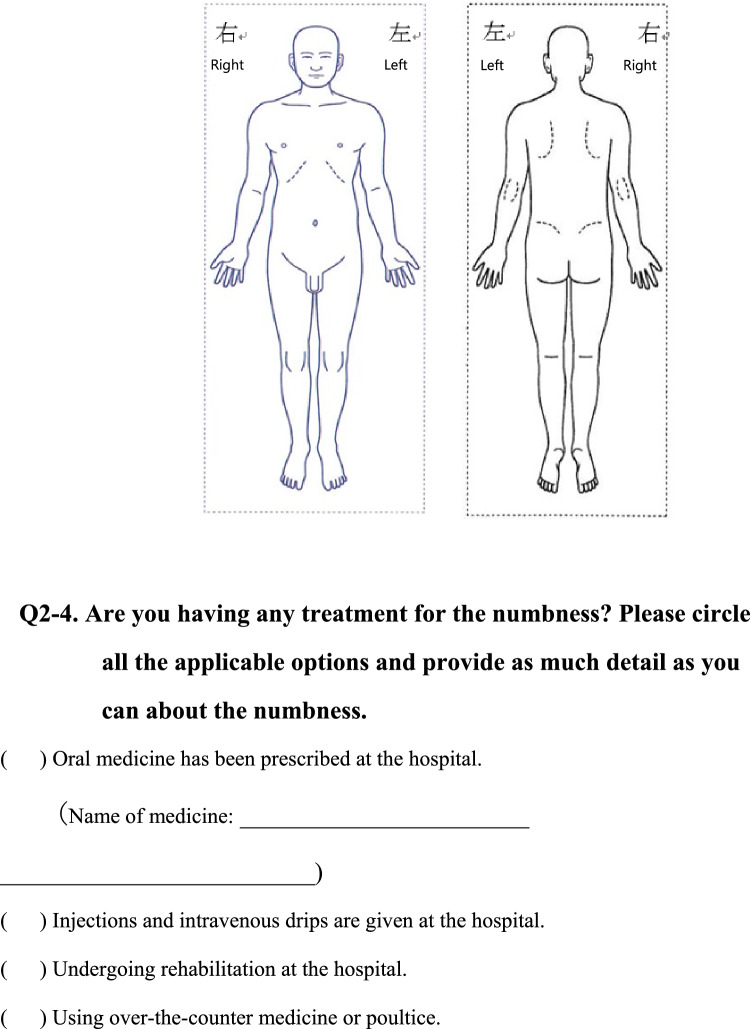

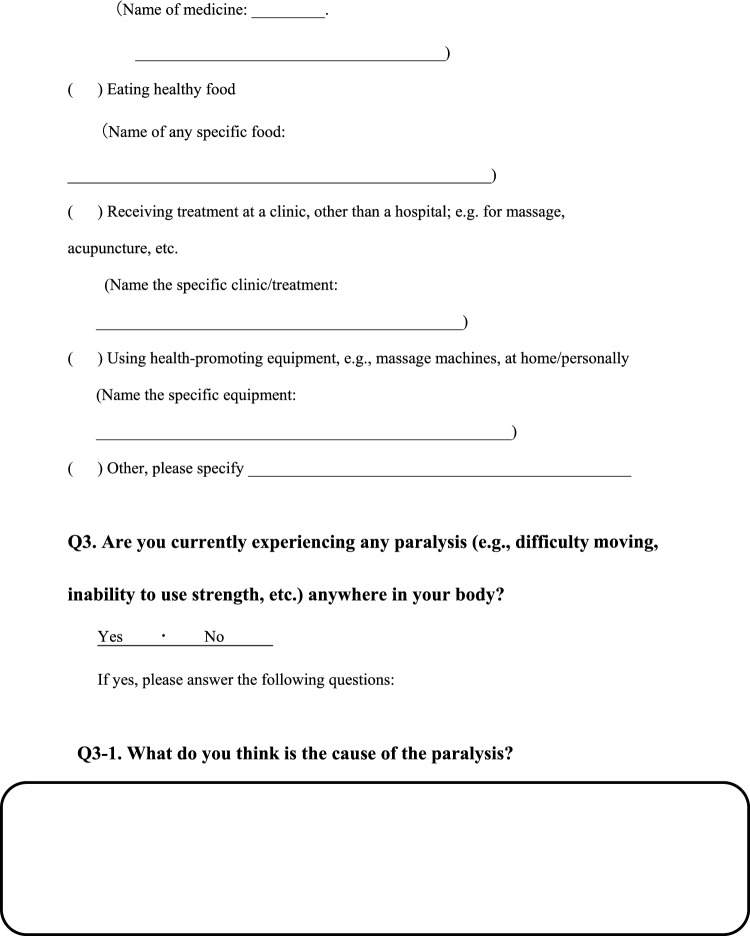

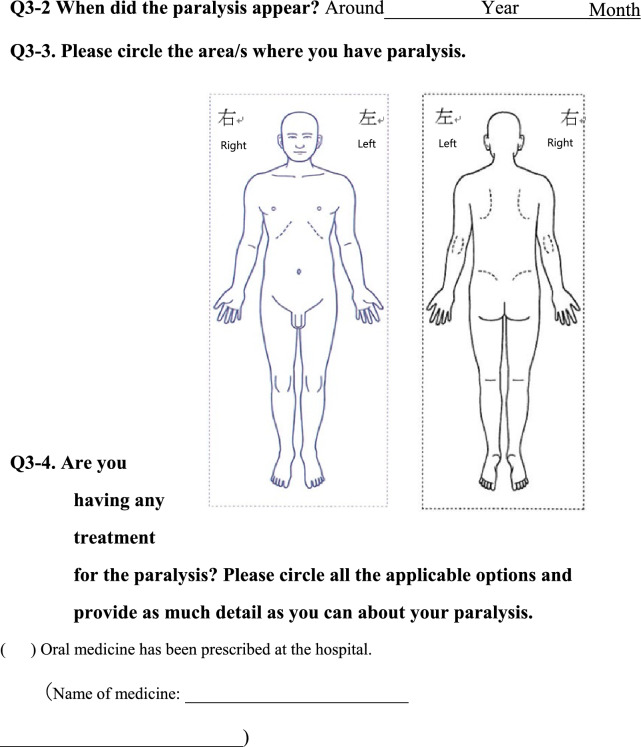

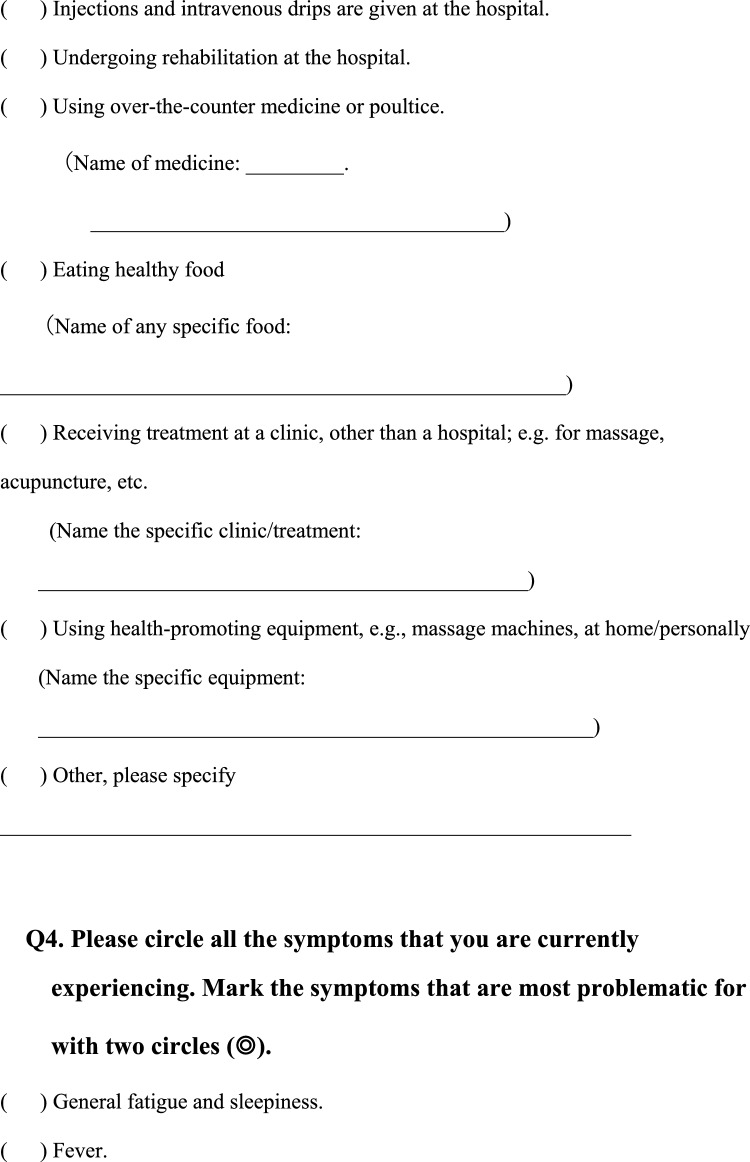

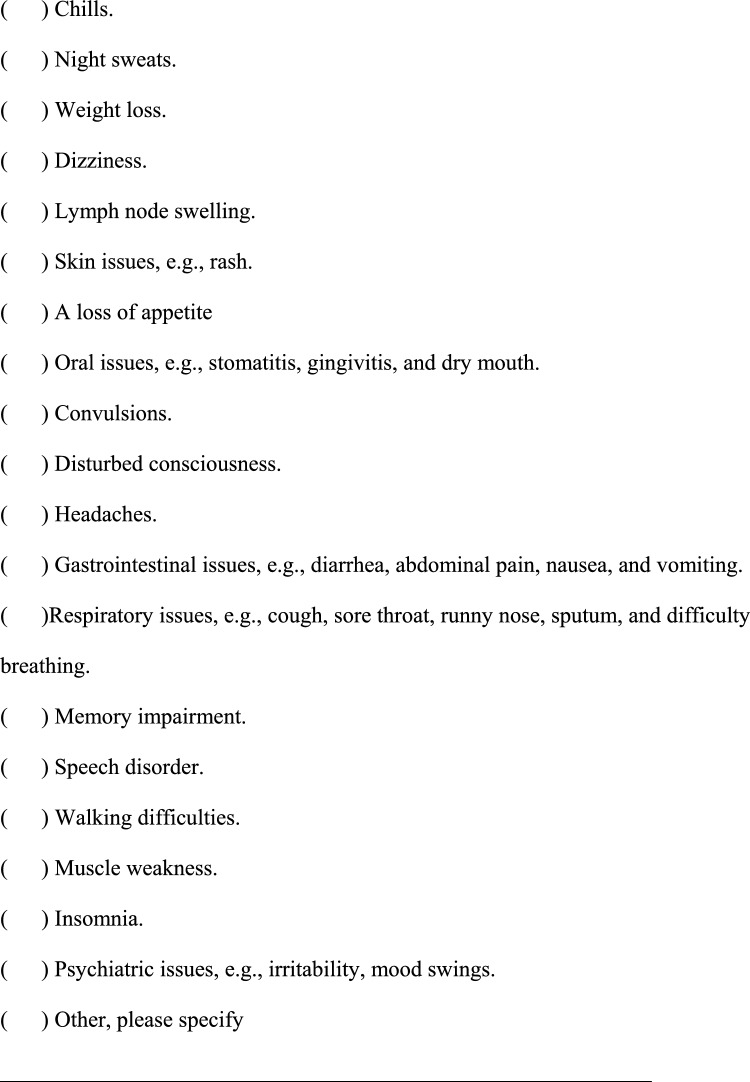

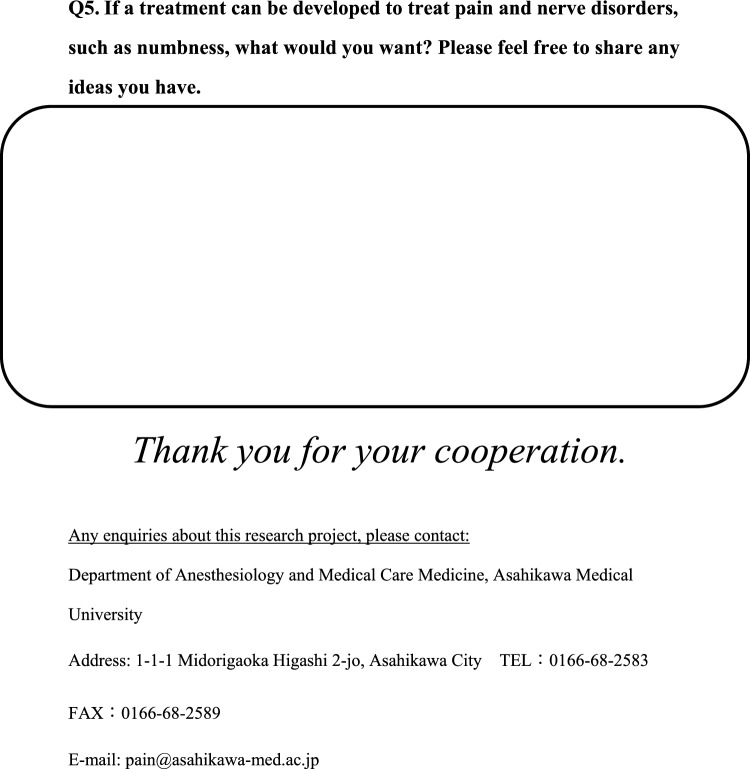
Japanese survey translated to English

### Statistical analysis

To compare the demographic and blood test results between those with and without HIV-related neuropathic pain, we used Student’s t-test or Mann–Whitney U test for continuous data and χ2 test or Fisher exact test for discrete variables. All p-values were considered statistically significant with P < 0.05. Data are presented as mean ± standard deviation for continuous variables, and percentage for categorical and sequential variables. All statistical analyses were performed by JMP statistical software (version 14.2; SAS Institute, Cary, NC).

## Results

A survey was distributed to 474 patients, of whom 270 chose not to participate. Consequently, data from 204 patients were included in the analysis. Of these, 97% of the patients were male (198 of 204 patients) and 97% of patients received treatment with HAART (198 of 204 patients)**.** Among the 204 patients included in this analysis, 72 patients responded “Yes” to Q1 (Table 1): “Do you currently have pain anywhere in your body?”. We excluded 40 patients who reported conditions with known causes of pain such as lumbar stenosis, disk herniation, trauma, hemophilic arthritis (hemarthrosis), and others in Q1-1 (Table 1): “What do you think is the cause of the pain?”. The remaining 32 patients (16%) who did not report conditions with known causes of pain were identified as possible HIV-related pain and included in the HIV-related pain group (Table 2). The characteristics of patients with and without HIV-related pain is summarized in Table 2. We found that 19 (59.4%) out of the 32 patients, identified their distal limbs (upper extremity or lower extremity or both) as the affected area, suggesting possible HIV-related peripheral neuropathy as a cause of their pain (19 out of 204, 9.3%). Additionally, nearly half of the patients who identified pain in distal limbs also experienced numbness (9 out of 19). The mean duration of pain in patients who reported HIV-related pain was approximately 4.5 years (54 months). Among these patients, several characteristics were significantly higher, including advanced age, presence and extended duration of numbness, and extended duration of AIDS, compared to patients without HIV-related pain. In contrast, there were no statistically significant differences in CD4 T-cell count, RNA load, treatment status, or duration of HAART between patients with and without HIV-related pain. Of note, all patients who reported pain were treated with HAART. Table 3 demonstrates the patient demographics of each of the 32 patients with HIV-related pain.

**Table 2:** Characteristics of patients with and without HIV-related pain

	HIV-related pain (n = 32)	No HIV-related pain (n = 172)	P value
Pain	32	–	–
Distal limbs as location of pain	19	–	–
Numbness in distal limbs	9	–	–
Duration of pain (months)	53.9 ± 5.27	–	–
Age (year)	52.2 ± 12.6	46.4 ± 11.5	0.0100
Sex(male/female)	31/1	167/5	1.0000
Numbness anywhere	16	16	< 0.001
Duration of numbness (months)	24.3 ± 53.1	3.81 ± 21.0	0.0003
Duration of HIV infection (months)	140 ± 105	122 ± 98.7	0.3450
CD4 T-cell count	554 ± 52.6	604 ± 308	0.3876
RNA load	3.44 ± 14.1	341 ± 3580	0.5942
Diagnosis of AIDS	16	56	0.0648
Duration of AIDS (months)	56.0 ± 78.8	27.5 ± 56.3	0.0161
Treatment with HAART	32	166	0.5927
Duration of HAART (months)	105 ± 79.8	90.2 ± 78.6	0.3371

*HIV* human immunodeficiency virus, *CD* cluster of differentiation, *RNA* ribonucleic acid, *AIDS* acquired immune deficiency syndrome, *HAART* highly active antiretroviral therapy

**Table 3:** Patient demographics

Patient no	Sex	Age (year)	Duration of pain (months)	Location of pain	Treatment	Presence of numbness anywhere
1	Male	39	216	LB, Trunk	None	None
2	Male	54	81	Neck, UB	None	Yes
3	Male	40	156	UE, LE, Neck, Gluteal	Medication, fomentation, local anesthetic injection	Yes
4	Male	63	1	LB	None	None
5	Male	70	216	Knee, LB	Fomentation	None
6	Male	47	4	UE, Neck, Occipital, LB	None	Yes
7	Male	49	Blank	Facial	Medication	None
8	Male	62	17	UE	None	None
9	Male	49	256	Knee, LB	Fomentation, massage tools	Yes
10	Male	44	5	UE, LB	Massage	Yes
11	Female	68	Blank	Blank	None	None
12	Male	68	Blank	Knee, LB	Fomentation	None
13	Male	49	96	LB	Medication	Yes
14	Male	39	36	UB	None	Yes
15	Male	68	36	LE	None	None
16	Male	59	4	UE, LE, LB	Fomentation, massage	Yes
17	Male	54	12	LE	Fomentation, massage	Yes
18	Male	58	108	UE, LE, LB	Fomentation	Yes
19	Male	33	12	LE	Heat treatment	Yes
20	Male	58	6	LE	None	None
21	Male	41	Blank	LE	Fomentation	Yes
22	Male	52	Blank	UE, LE	Fomentation	Yes
23	Male	85	2	LE	Massage, massage tools	None
24	Male	48	50	UE, LE	Massage	Yes
25	Male	31	2	UE	None	None
26	Male	32	26	Head	Medication	Yes
27	Male	52	12	LE	None	None
28	Male	55	2	UE	None	Yes
29	Male	37	0	LE, Trunk	Medication	None
30	Male	66	96	UB	Exercise	None
31	Male	53	1	UE	None	None
32	Male	48	4	LE	None	None

Duration of numbness (months)	Duration of HIV infection (months)	CD4 T-cell count	RNA load	Diagnosis of AIDS	Duration of AIDS (months)	Treatment with HAART	Duration of HAART (months)
0	Blank	376	0	Blank	0	Yes	42
2	371	312	0	Yes	250	Yes	248
216	391	805	0	None	0	Yes	319
0	185	566	0	Yes	185	Yes	185
0	170	410	0	Yes	170	Yes	167
Blank	179	668	0	None	0	Yes	126
0	217	560	0	None	0	Yes	121
0	148	621	0	None	0	Yes	139
11	144	551	0	None	0	Yes	139
2	140	859	0	None	0	Yes	104
0	134	404	0	Yes	134	Yes	132
0	332	293	0	Yes	124	Yes	116
96	131	686	0	None	0	Yes	37
36	283	474	0	None	0	Yes	143
0	125	221	0	Yes	125	Yes	121
4	98	290	70	Yes	31	Yes	31
24	99	741	0	None	0	Yes	82
Blank	85	451	0	Yes	84	Yes	74
36	60	802	0	None	0	Yes	55
0	70	271	0	Yes	71	Yes	70
180	56	831	0	Yes	57	Yes	55
Blank	55	949	0	None	0	Yes	50
0	51	457	0	None	0	Yes	49
50	51	452	0	Yes	50	Yes	49
0	55	730	40	None	0	Yes	35
45	49	922	0	None	0	Yes	38
0	36	714	0	None	0	Yes	10
2	100	711	0	Yes		Yes	97
0	22	314	0	Yes	22	Yes	21
0	356	872	0	Yes	260	Yes	332
0	27	149	0	Yes	28	Yes	27
0	145	277	0	Yes	145	Yes	142

*HIV* human immunodeficiency virus, *CD* cluster of differentiation, *RNA* ribonucleic acid, *AIDS* acquired immune deficiency syndrome, *HAAR*T highly active antiretroviral therapy, *LB* lower back, *UB* upper back, *UE* upper extremity, *LE* lower extremity

## Discussion

By analyzing a clinical survey and medical records of 204 Japanese patients with HIV infection, we found that the prevalence of HIV-related pain was 16% and patients with possible HIV-related peripheral neuropathic pain was 9.3%. Among these patients, age, presence and duration of numbness, and duration of AIDS was significantly higher than in patients without HIV-related pain. Further, we found that approximately 60% of the patients who reported pain identified their distal limbs as the affected area, suggesting possible HIV-related peripheral neuropathy as a cause of their pain. Additionally, nearly half of the patients who identified pain in distal limbs also experienced numbness. However, we acknowledge that not all of these patients meet the diagnostic criteria for peripheral neuropathy, as a self-reported survey alone cannot establish this diagnosis.

To the best of our knowledge, this is the first study to investigate the prevalence and characteristics of patients with HIV-related pain with a focus on peripheral neuropathic pain in Japanese patients since the widespread adoption of HAART in 1996. The prevalence of overall neuropathy among patients with HIV in the United States, derived from 25 studies, varied from 1.2% to 69.4% [21]. Further, the prevalence of HIV-related peripheral neuropathy has been indicated as 6.9–10% in a systematic review of epidemiological studies [11], while the reports vary between 30 and 50% of HIV-positive patients [17], and one study reported a high prevalence of distal sensory polyneuropathy at 57% in the HAART era [22]. Another study indicated that while the prevalence of HIV-related peripheral neuropathy in patients who have not received HAART was 29%, the number increased to 38% for patients at various stages of the disease [23]. Accordingly, HIV-related neuropathic pain is identified to be associated with the virus itself, opportunistic infections, and neurotoxic effects of HAART, known as ATN [13, 16, 24]. A nationwide survey conducted in Japan before the introduction of HAART in 1996 showed that 2.6% of the 578 individuals with AIDS reported peripheral neuropathy [18]. Conversely, our study showed the prevalence of HIV-related pain was 16% with approximately 60% of these patients identifying their distal limbs as the affected area and nearly half of the patients who identified pain in distal limbs also experienced numbness, suggesting possible HIV-related peripheral neuropathy. All patients with HIV-related pain were treated with HARRT.

There are several potential reasons that the prevalence rate of HIV-related pain and possible HIV-related peripheral neuropathic pain was lower in our study compared to studies in the US. First, the smaller sample size and self-reported nature of the clinical survey may have led to less representation and underreporting, as other studies typically include clinical diagnoses, different patient behavior and reporting standards. We believe that the limited awareness and recognition of HIV-related pain among both patients and healthcare providers in Japan may contribute to underdiagnosing, low treatment rate, and further limiting treatment options due to insufficient research. Furthermore, our study revealed that several patient characteristics were significantly higher in patients with HIV-related pain, such as advanced age, presence and extended duration of numbness, and extended duration of AIDS compared to patients without HIV-related peripheral neuropathic pain. This trend of advanced age and extended duration of chronic systemic disease is similar to that seen in diabetic neuropathy and chemotherapy-induced peripheral neuropathy, which both have similar manifestations to HIV-related peripheral neuropathy. Moreover, advanced age noted in our results was consistent with one of the risk factors suggested as HIV-related peripheral neuropathy in a previous report, while others included were treatment with HAART, low CD count, elevated plasma HIV-1 RNA viral load, advanced age, and history of substance abuse [17]. In contrast, there were no significant differences seen in our study for CD4 T-cell count, RNA load, treatment status, or duration of HAART between patients with and without HIV-related pain, although this is likely due to the high rate of treatment of HAART (97% of all patients in our study).

Finally, this study has several potential limitations. Our study faces inherent challenges associated with survey-based research, including a high dropout rate, potential response bias, and reliance on self-reported data. Self-reported data can be subjective and prone to inaccuracies such as underreporting, recall bias, or misinterpretation of survey questions, and selection bias as only individuals who opted to participate were included. Furthermore, the self-reported nature of the questionnaire complicates the determination of whether the identified pain was truly HIV-related. Specifically, the 32 patients that we identified as HIV-related pain were those who did not report conditions with known causes of pain, while not all explicitly attributed their pain to HIV. Nonetheless, we considered it important to include all 32 patients in the analysis for possible HIV-related pain, regardless of whether they identified HIV as the cause because HIV-related pain is not well recognized among Japanese patients or healthcare providers, likely contributing to its underdiagnosis and undertreatment. Additionally, it is uncommon for HIV patients to attribute their pain to HIV, further supporting the inclusion of these cases in our analysis. Moreover, the generalizability of this study may be constrained by the small sample size and reliance on data from two institution, as regional variations in healthcare access, clinical practices, and patient demographics could impact the applicability of the findings to broader populations. However, it is important to highlight that our study analyzed a meaningful sample size of over 200 patients, providing valuable insights into the study population. Despite these limitations, our study provides meaningful context by aligning with national epidemiological data. Specifically, 97% of our participants were male, which corresponds closely to the gender distribution of patients with HIV infection and AIDS in Japan reported in the 2023 national epidemiological data from the Japanese Ministry of Health, Labour and Welfare. According to this report, the gender distribution was 649 males versus 20 females (97.0% male) for individuals with HIV and 282 males versus 9 females (96.9% male) for those with AIDS [25]. This consistency strengthens the robustness of our discussion by providing a contextual foundation for interpreting our study findings within the broader national landscape. Furthermore, this highlights the importance of tailoring interventions and healthcare services to address the predominantly male demographic affected by HIV in Japan.

## Conclusion

In this prospective multi-center cross-sectional study, we found that the prevalence of HIV-related pain was 16% by analyzing a survey and medical records of 204 Japanese patients with HIV infection and patients with possible HIV-related peripheral neuropathic pain was 9.3%. Among these patients, age, presence and duration of numbness, duration of AIDS was significantly higher than in patients without HIV-related pain. To further enhance the understanding of HIV-related pain and peripheral neuropathic pain, multi-center and nationwide studies are warranted to validate and expand upon our findings, including the prevalence and risk factors of these conditions. Limited awareness and recognition of HIV-related pain among both patients and healthcare providers in Japan, such as pain clinicians, anesthesiologists, and infectious disease specialists, likely contributes to underdiagnosis and low treatment rates. Addressing these gaps through comprehensive studies is essential to improving diagnosis, treatment strategies, and overall patient care.

## Data Availability

The datasets for the current study are not publicly available due to institutional restrictions but may be available from the corresponding author on reasonable request, subject to institutional and ethical approvals.
